# To Graduates of Rush Medical College

**Published:** 1867-07

**Authors:** R. L. Rea

**Affiliations:** 119 Clark Street, Room 13, Chicago


### *To Graduates of Rush Medical College.*

EDITOR CHICAGO MEDICAL JOURNAL:—I would like to obtain, as far as possible, a complete list of the graduates of Rush Medical College and their locations, and would hereby request *all* graduates of it to forward me their address; and, as this may not reach all, those sending are requested to forward the names and residences of any others with which they may be acquainted. I hope this request may be promptly complied with.

It was proposed, when our new college was projected, that a grand alumni meeting should be held at the dedication, about the 1st of October next. How do you all feel about it? It would give the Faculty very great pleasure to see you all together, and have you participate in the grateful task of dedicating to medical science the beautiful temple now erecting.

Address R. L. REA,
*119 Clark Street, Room 13, Chicago.*

